# Next-generation sequencing identifies articular cartilage and subchondral bone miRNAs after ESWT on early osteoarthritis knee

**DOI:** 10.18632/oncotarget.11331

**Published:** 2016-08-17

**Authors:** Jai-Hong Cheng, Ching-Jen Wang, Shi-Hao Su, Chien-Yiu Huang, Shan-Ling Hsu

**Affiliations:** ^1^ Center for Shockwave Medicine and Tissue Engineering, Kaohsiung Chang Gung Memorial Hospital and Chang Gung University College of Medicine, Kaohsiung, Taiwan; ^2^ Department of Orthopedic Surgery, Kaohsiung Chang Gung Memorial Hospital and Chang Gung University College of Medicine, Kaohsiung, Taiwan; ^3^ Medical Research, Kaohsiung Chang Gung Memorial Hospital and Chang Gung University College of Medicine, Kaohsiung, Taiwan

**Keywords:** osteoarthritis, extracorporeal shock wave therapy, miRNA, next-generation sequencing

## Abstract

Extracorporeal shockwave therapy (ESWT) has shown chondroprotective effects on the initiation of the osteoarthritis (OA) changes of the rat knee. This study evaluated 69 significant expressed profiles of microRNA (miRNA) in the articular cartilage and subchondral bone after ESWT. There were 118 target genes identified for miRNAs of interest in articular cartilage and 214 target genes in subchondral bone by next generation sequencing (NGS). In principal component analysis (PCA), the relationships of miRNA expression in bone and cartilage were improved after ESWT. Global functional annotation showed that predicted targets were involved in cartilage development, inflammatory and immune response, ion binding, angiogenesis, cell adhesion, cell cycle, transcription and translation, gene expression, NTP binding, signal transduction, collagen fibril organization, apoptotic process, chondrocyte differentiation, cell differentiation, bone development as well as cell proliferation. The miRNAs profile and the target genes were comprehensively surveyed and compared in articular cartilage and subchondral bone of early OA knee before and after ESWT. Our study represents the direct assessment to date of miRNA expression profiling in early OA articular cartilage and subchondral bone. The results provide insights that could contribute to the development of new biomarkers and therapeutic strategies for OA changes and the treatment with ESWT.

## INTRODUCTION

MicroRNAs (miRNAs) are single-stranded, small noncoding RNA molecules of 18 to 24 nt in length that negatively regulate the expression of target genes in a post-transcriptional manner. Hundreds of miRNAs have been found in various organisms, and many miRNAs are evolutionarily conserved. Moreover, one third of all mammalian mRNAs seem to be under miRNAs regulation, suggesting it has an essential role in regulating gene expression [1]. Recent evidences have also indicated that these small RNA molecules play a role in the biological processes as well as pathogenesis of human disorders such as birth defects [2], apoptosis/proliferation [3, 4], glucose/lipid metabolism [5], cell development [6, 7], and cancers [2, 8, 9]. The expression profiles of miRNAs are effective for classification of human disease. Current methodologies have been developed and applied successfully in miRNAs profiling, including microRNA arrays [10]. Currently available clinical treatments of OA knee tend to be unsatisfactory. Novel targets in OA include genes that are involved in OA pathophysiology, which have been discovered using gene network, epigenetic and miRNAs approaches. Studies on different miRNAs have revealed that they have pro-inflammatory and catabolic/anabolic roles in the pathophysiology of OA [11]. MiRNAs have emerged as important modulators in the development, tissue homeostasis and diseases as they may be targets for articular cartilage tissue engineering and regenerative medicine. With more studies on miRNAs, the relationship between miRNAs and OA has become closer than once predicted.

Extracorporeal shockwave therapy (ESWT) has been used in the treatment of soft tissue and bone related musculoskeletal disorders for over 20 years. ESWT has shown effectiveness in many orthopedic disorders including soft tissue tendinopathy and non-union of long bone fractures [12, 13]. In addition, many studies including ours reported positive effects of ESWT in various arthritic joints in animals [14-18]. Our previous studies demonstrated that ESWT is chondroprotective in the initiation of OA changes of the knee [19, 20], and induces regression or retardation of established OA changes of the knee in rats [21]. Many studies including ours reported a dose-related effect of ESWT in bone [22], tendon [23], epigastric skin flap [24], tenocyte [25], osteoarthritis of the knee [26] and cells [27]. ESWT is a new non-invasive therapeutic modality with effectiveness, convenience and safety. ESWT has the potential of replacing surgery in many orthopedic disorders without the surgical risks. The exact mechanism of shockwave therapy remains unknown. In animal experiments, ESWT induces a cascade of biological responses and molecular changes including the ingrowth of neovascularization and promotion of angiogenetic growth factors leading to the improvement in blood supply and tissue regeneration. There is a great potential for translational research and development in the armamentarium of extracorporeal shockwave technology.

MiRNAs profiling in human cartilage and bone has been performed, and miRNAs targets have been identified with relevance to OA changes [1, 6, 28, 29]. Several miRNAs are demonstrated that associated with OA development and modulation such as miR-18a (chondrocyte differentiation), miR-27b (controlling the expression of MMP-13), miR-34a (prevention of cartilage degradation), miR-140 and miR-222 (controlling cartilage homeostasis), miR-146 (promotion of inflammatory OA), miR146a (OA cartilage pathogenesis), miR-675 (cartilage repair) [30, 31]. With the development of NGS technology, changes in the expression levels of thousands of genes and miRNAs can be examined simultaneously, and integral analysis of the dysregulated genes can be performed to obtain information regarding pathogenic mechanisms of OA at the cellular level, animal model and human disease [32, 33]. Furthermore, NGS data can be used to discover novel molecular diagnostic markers and therapeutic targets [34]. However, miRNAs controlling gene expression profiles of OA treated with EWST in subchondral bone that are associated with cartilage degeneration remain unknown. The aims of this project, is to identify and characterize the gene expression profile of miRNAs in early OA knee treated with ESWT to articular cartilage and subchondral bone and to determine their role and the molecular mechanism of ESWT in OA.

## RESULTS AND DISCUSSION

### Validation of the rat model of early OA

**Figure 1:**
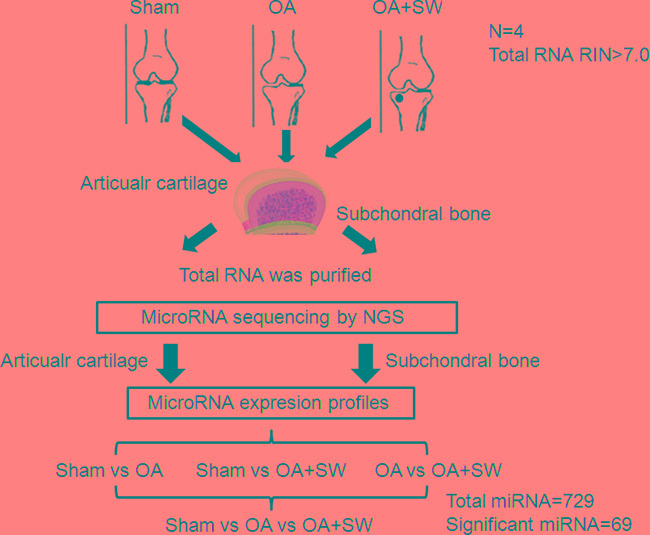
Experimental design Twelve rats were divided in three groups such as sham, osteoarthritis (OA) and OA with shockwave (SW). The miRNA from sham, OA and OA+SW cartilage and bone samples were purified and then were sequencing by NGS. Four comparisons were used to analysis the expression of miRNA. The 729 miRNAs were analysis and 69 miRNAs were significant in our experiment. The black circle was the site for shockwave treatment.

**Figure 2:**
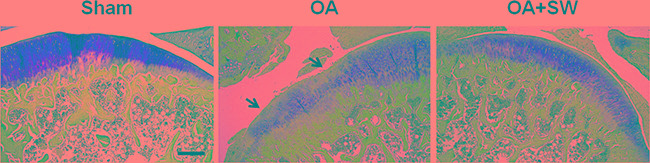
The histologic analysis of cartilage and subchondral bone in a rat model of early OA Safranin O staining of sagittal sections of the tibia medial compartment and proteoglycan of cartilage (red) and subchondral bone (blue) was shown. Black arrows indicated loss of proteoglycan at 4 weeks post-surgery. Scale bar, 50 μm.

The progression and early histological events of OA disease has been observed in a time dependent manner in the animal model [35, 36]. We used an anterior cruciate ligament transection in combination with medial meniscectomy (ACLT+MM) effectively induced OA-like early changes in the cartilage, as reported previously [35, 36]. ESWT was applied on the subchondral bone of the medial tibia condyle of the left knee after surgery. Then we scarified the rats at 4 weeks after surgery and collected the articular cartilage and subchondral bone to analysis (Figure 1). In the Safranin O staining, the articular surface damage and fibrillation was observed around 4 weeks post-surgery in OA group (Figure 2). However, in ESWT group, the articular surface was not obvious damage and only slight fibrillation on the surface. At this stage, we collected the small RNA sample from articular cartilage and subchondral bone to identify the specific miRNAs which activated or supressed by ESWT on early OA treatment.

### Identification and characterization of miRNAs in subchondral bone and cartilage tissues

**Figure 3:**
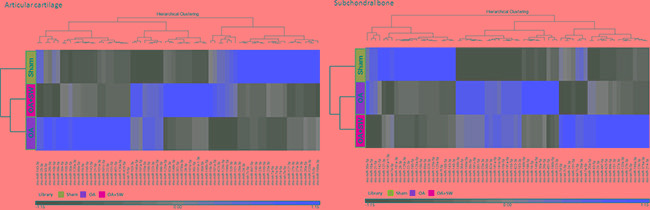
Hierarchical clustering of the miRNA identified in each expression of library pattern from subchondral bone The miRNAs were analysis from subchondral bone of sham (blue), OA (orange) and OA+SW (yellow). The color in clustering was greed=down regulation, and red= up regulation. Total miRNA was 69.

There are several advanced methods to study the profile of miRNAs in OA from human and animals such as microarray, Solexa-based deep sequencing and next generation sequencing (NGS) [37-39]. Recently, Dr. Desjardin showed 609 and 622 miRNAs expression profiles in cartilage and bone, respectively, including 282 and 293 putative new miRNAs in cartilage and bone from equine [38]. Dr. Sun identified expression profiles of miRNAs of articular cartilage from rat femoral head cartilage. They made it possible to highlight 310 known miRNAs including 86 novel miRNAs candidates [39]. Current, we used NGS to analysis the miRNA expression profiles from articular cartilage and subchondral bone in normal, OA and OA+shockwave (SW). RIN of the total RNA between 7 and 8 were observed for samples of cartilage and subchondral bone. In order to show the output data by miRSeq and to analysis the performance of miRSeq, we used small RNA NGS data of the six libraries (Both cartilage and subchondral bone are sham, OA and OA+SW groups) to analysis by miRSeq [40]. We elaborated a set of 729 expressed miRNAs in cartilage and subchondral bone. Sixty-nine miRNAs were found and significant in both tissues (Transcript per million (TPM) > 10000) [40]. We employed a hierarchical clustering based on the variation of each miRNA expression across the experimental groups (sham, OA and OA+SW) in articular cartilage and subchondral bone (Figure 3). We found the level of miRNAs expression were obviously different between articular cartilage and subchondral bone after ESWT. For example, rno-miR-199a-3p was decreased in cartilage of OA+SW group, however, was highly increased in bone. In the Figure 3, the expression level of each miRNA was identified by using the TaqMan® assays. These results showed ESWT stimulated or inhibited expression of several specific miRNA between cartilage and subchondral bone comparing with sham, OA and OA+SW groups.

### Differential miRNAs expression analysis

Expression levels of miRNA in subchondral bone and articular cartilage from sham, OA and OA+SW were compared to determine the potential role of miRNAs in early OA physiopathology and shockwave treatment. This analysis highlighted 14 miRNAs differentially expressed in articular cartilage and 12 miRNAs in subchondral bone (Table 1: articular cartilage and Table 2 : subchondral bone). In articular cartilage, 7 miRNAs, 8 miRNAs and 4 miRNAs were found to be increased but, 5 miRNAs, 5miRNAs and 10 miRNAs were decreased in sham vs OA, sham vs OA+SW and OA vs OA+SW groups, respectively (Table 1). In the subchondral bone, 4 miRNAs, 3 miRNAs and 3miRNA were increased but, 6 miRNAs, 9 miRNA and 9 miRNAs were decreased in sham vs OA, sham vs OA+SW and OA vs OA+SW groups, respectively (Table 2).

**Table 1:** The profiles and expressions of miRNA in articular cartilage with different comparisons

	Sham vs OA	Sham vs OA+SW	OA vs OA+SW	miRNA Expression
miR-ID	rno-miR-451-5p	rno-miR-451-5p	rno-miR-378a-3p	UP regulation
	rno-miR-191a-5p	rno-miR-378a-3p	rno-miR-191a-5p	
	rno-miR-30e-5p	rno-miR-191a-5p	rno-miR-181a-5p	
	rno-miR-22-3p	rno-miR-181a-5p	rno-miR-125a-5p	
	rno-miR-21-5p	rno-miR-125a-5p		
	rno-miR-16-5p	rno-miR-30e-5p		
	rno-miR-10b-5p	rno-miR-21-5p		
		rno-miR-16-5p		
miR-ID	rno-miR-199a-3p	rno-miR-199a-3p	rno-miR-451-5p	Down regulation
	rno-miR-181a-5p	rno-miR-143-3p	rno-miR-199a-3p	
	rno-miR-125a-5p	rno-miR-140-3p	rno-miR-143-3p	
	rno-miR-140-3p	rno-miR-27b-3p	rno-miR-140-3p	
	rno-miR-27b-3p	rno-miR-10b-5p	rno-miR-30e-5p	
			rno-miR-27b-3p	
			rno-miR-22-3p	
			rno-miR-21-5p	
			rno-miR-16-5p	
			rno-miR-10b-5p	

**Table 2:** The profiles and expressions of miRNA in subchondral bone with different comparisons

	Sham vs OA	Sham vs OA+SW	OA vs OA+SW	miRNA Expression
miR-ID	rno-miR-191a-5p	rno-miR-191a-5p	rno-miR-191a-5p	UP regulation
	rno-miR-92a-3p	rno-miR-181a-5p	rno-miR-181a-5p	
	rno-miR-143-3p	rno-miR-92a-3p	rno-miR-92a-3p	
	rno-miR-10a-5p			
miR-ID	rno-miR-181a-5p	rno-miR-143-3p	rno-miR-143-3p	Down regulation
	rno-miR-142-5p	rno-miR-142-5p	rno-miR-142-5p	
	rno-miR-140-3p	rno-miR-140-3p	rno-miR-140-3p	
	rno-miR-30e-5p	rno-miR-30e-5p	rno-miR-30e-5p	
	rno-miR-27b-3p	rno-miR-27b-3p	rno-miR-27b-3p	
	rno-miR-21-5p	rno-miR-22-3p	rno-miR-22-3p	
		rno-miR-21-5p	rno-miR-21-5p	
		rno-miR-10a-5p	rno-miR-10a-5p	
		rno-miR-10b-5p	rno-miR-10b-5p	

### Principal component analysis and relationship of miRNAs

**Figure 4:**
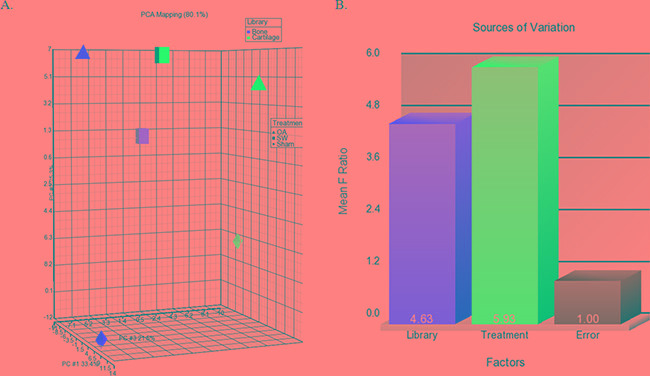
The profile of overall miRNAs expression changes **A.** The differential expression levels of miRNA in library (bone and cartilage) versus treatment groups (sham, OA and OA+SW). Each shape in the 3D visualization represents a group of miRNAs. Principal component analysis (PCA) captured 80.1% of the variation observed in the experiment in the first three principal components (PC). **B.** The source of the variation plot showed that the expression of miRNA in treatment groups of factor accounted for major of the variation.

**Figure 5:**
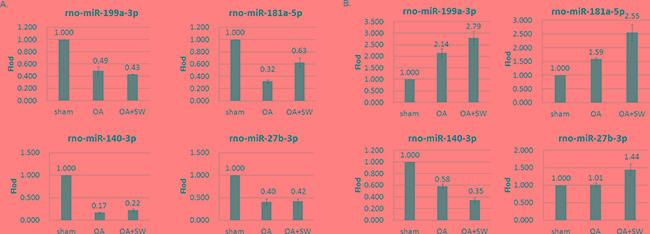
MiRNA expression patterns were evaluated by Quantitative RT-PCR The rno-miR-199a-3p, rno-miR-181a-5p, rno-miR-140-3p and rno-miR-27b-3p were measured from articular cartilage **A.** and subchondral bone **B.** The expression profiles of each miRNA matched the tested probes at least three times repeat. The values were the mean and SEM of the miRNA expression levels in 4 animals, as determined by ΔCt analysis, normalized to GAPDH expression, and relative to the expression levels of sham control. ** p<0.01 (versus sham control).

The 3D visualization of the relationships was illustrated between the expressed miRNAs of library (Subchondral bone and cartilage) and treatment groups (Sham, OA and OA+SW) using principal component analysis (PCA) (Figure 4A). The clustered miRNAs were in separate areas of the 3-dimentional visualization, indicating a clear difference in the molecular composition between subchondral bone and cartilage. The difference of miRAN expressions also were observed in treatment groups of sham and OA. However, the relationships of miRNA expression in bone and cartilage were improved after SW treatment. The source of the variation plot showed that the treatment groups of factor accounted for major of the variation in miRNA expression (Figure 4B). The level of expressed miRNAs of interest, rno-miR-199a-3p, rno-miR-181a-5p, rno-miR-140-3p and rno-miR-27b-3p, were confirmed by quantitative RT-PCR in articular cartilage and subchondral bone (Figure 5A and 5B). It have been reported that miR-140 is speciﬁcally expressed in cartilage tissue to activate or supress PDGF, SOX9, Sp1, aggrecan, VEGF and HDAC4 [28, 41]. Several miRNAs including miR-140, miR-199a, mir-193 and mir-29a/29b control the anabolic and catabolic regulation in cartilage [42]. In human OA chondrocytes, miR-27b controls the expression of matrix metalloproteinase 13(MMP13) in OA treatment [43]. MiR-181a is reported to control osteopontin expression in cancer cell and inhibit expression in bone marrow-derived mesenchymal stem cells to maintain bone remodeling balance [44, 45].

### Prediction and analysis the effective genes of miRNAs in articular cartilage and subchondral bone before and after ESWT

Predicted target genes of differentially expressed miRNAs in articular cartilage and subchondral bone were identified using the database TargetScan [46]. In articular cartilage, the increased miRNAs (7, 8 and 4 miRNAs) of sham vs OA, sham vs OA+SW and OA vs OA+SW were predicted to inhibit the expression of the 33, 98 and 88 genes, respectively (Supplementary Data 1). In contrast to the decreased miRNAs (5, 5 and 10 miRNAs) of sham vs OA, sham vs OA+SW and OA vs OA+SW were predicted to activate the 14, 15, and 18 genes, respectively (Supplementary Data 2).

The functional annotation miRNAs in subchondral bone were also predicted target genes. The increased miRNAs (4, 3, and 3 miRNAs) of shma vs OA, sham vs OA+SW, and OA vs OA+SW were predicted to inhibit the 128, 167, and 13 genes, respectively (Supplementary Data 3). Further, the decreased miRNAs (6, 9 and 9 miRNAs) of sham vs OA, sham vs OA+SW and OA vs OA+SW were predicted to activate the 27, 43 and 14 genes, respectively (Supplementary Data 4).

### Classification and functional annotation of miRNAs predicted targets

**Figure 6:**
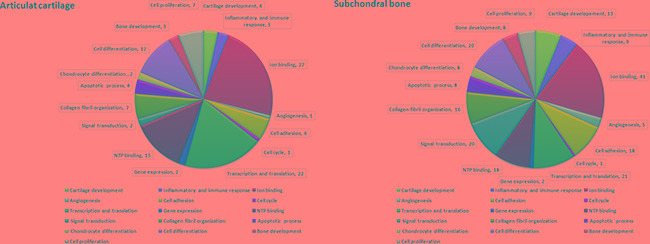
Molecular functions of predicted target genes of miRNAs differentially expressed in articular cartilage and subchondral bone The diagrams of **A.** articular cartilage and **B.** subchondral bone showed the proportion of modulated miRNAs target genes belonging to the most significant functional annotations derived from the UniProt database.

We used UniProt database (http://www.uniprot.org/) analyses to perform more detailed functional information regarding the predicted genes. In OA knee of equine study, there are about 2400 putative target genes to be identified for miRNAs. The each miRNA is correlated with 42 to 250 target genes in cartilage and with 51 to 250 target genes in bone [38]. In our study, there were 118 target genes identified for miRNAs of interest in articular cartilage and 214 target genes in subchondral bone. Global functional annotation showed that predicted targets were involved in cartilage development, inflammatory and immune response, ion binding, angiogenesis, cell adhesion, cell cycle, transcription and translation, gene expression, NTP binding, signal transduction, collagen fibril organization, apoptotic process, chondrocyte differentiation, cell differentiation, bone development as well as cell proliferation (Figure 6A: articular cartilage; Figure 6B: subchondral bone). These results showed the profile of miRNAs and target genes between the relationship of sham, OA and OA+SW. Particularly, our data suggested ESWT induced expression of miRNA to control the genes which correlated with cartilage development and bone remodeling in OA treatment.

## MATERIALS AND METHODS

### Animals

All the animals were treated humanely according to the guidelines provided in the Guide for the Care and Use of Laboratory Animals, published by the National Institute of Health. All animals were housed under standard conditions. The Division of Laboratory Animal Resources at Chang Gung Memorial Hospital (CGMH), Kaohsiung Medical Center, administered veterinary care to the rodents. This study was subjected to the approval of the Institutional Animal Care and Use Committee (IACUC) at CGMH (Number: 2013111901).

### OA knee of rat model

This project was performed in 30 male Sprague-Dawley rats (each group for 8 rats) of 10-week old with body weight ranging from 275 mg to 315 mg. The animals were sedated with intra-peritoneal phenobarbital injection (50 mg/Kg body weight). The left knee was prepared and draped in surgically sterile fashion. A straight anterior skin incision was made and the knee joint was opened through medial parapatellar arthrotomy. The anterior cruciate ligament was transected with a scalpel. Medial meniscectomy was performed by excising the entire medial meniscus. The knee was irrigated and the wound was closed in routine fashion. The animals were returned to the housing cages under the care of the veterinarian.

### ESWT application

The animals in the ACLT plus ESWT group was received ESWT at 1 wk after knee surgery when the surgical wound healed. The source of shockwave was from a focused ESWT machine (Piezoson 100 plus, Richard Wolf GmbH). The focus area for SW was on the subchondral bone of the medial tibia condyle of the left knee at 0.5 cm below the joint line and 0.5 cm from the medial skin surface by ultrasound guiding (Toshiba SSA-660A Xario Ultrasound) [20]. The surgical lubricate was used on the skin in contact with the shockwave device. Each knee was treated with 800 impulses of shockwave at 0.22 mJ/mm^2^ energy flux density in a single session and frequency was 4 Hz. After ESWT, the animals were returned to the housing cage for routine care and observation.

### Specimen processing

The animals were sacrificed at 4 weeks post-ESWT. Twelve animals were used for time point in each treatment group. The specimens were used for histological analysis, miRNA NGS analysis and quantitative RT-PCR. Following disarticulation of the right knee joints, the tibias were cleaned and washed with physiologic saline. The gross appearance of the proximal tibia was recorded using a digital camera, and the distal section of each tibia will fix in 4% paraformaldehyde in phosphatebuffered saline (PBS) for 24 h. The samples were then decalcified with 20% EDTA (pH 7.4) for 3 weeks at 4°C and the medium was changed every 3 days. Following dis-articulation of the right knee joints of the other ten animals, the tibias were rapidly fractured and then frozen in liquid nitrogen for cartilage, subchondral bone separation and total RNA extraction.

### Total RNA extraction

Total RNA from cartilage and subchondral bone of sham (4 rats), OA (4 rats) and OA with ESWT (4 rats) were extracted by Trizol® Reagent (Invitrogen, USA) according to the instruction manual. Purified RNA was quantified at OD260nm by using a ND-1000 spectrophotometer (Nanodrop Technology, USA) and qualitated by using a Bioanalyzer 2100 (Agilent Technology, USA) with RNA 6000 labchip kit (Agilent Technologies, USA).

### Library preparation, sequencing and analysis

All procedures were carried out according to the manufacture's protocol from Illumina. Library constructions of each pooling RNA samples (sham, OA and OA+SW) were used by Agilent's SureSelect Strand Specific RNA Library Preparation Kit for 75SE bp (Single-End or Paired-End) sequencing on Solexa platform (Figure 1). The sequence was directly determined using sequencing-by-synthesis technology via the TruSeq SBS Kit. Raw sequences were obtained from the Illumina Pipeline software bcl2fastq v2.0 and expected to generate 40M (million reads or Gb) per sample. Trimmomatics was implemented to trim or remove the reads according to the quality score. Principal component analysis was performed to test dissociation of miRNA expression between the library tissues (bone and cartilage) and treatment groups (sham, OA and OA+SW). The profile of differentially expression of miRNA underwent pathway enrichment analysis with Partek from Partek Incorporated (Saint Louis, MO, USA).

### Histomorphological examination

The animals were sacrificed at 4 weeks post-surgery. The knee specimens including the articular cartilage and the subchondral bone of proximal tibia and distal femur were harvested. The specimens were decalcified and fixed in paraffin, and cut into 5-um thick sections using microderme and stained with Safranin-O.

### Quantitative RT-PCR of mature miRNAs

The reverse transcription reaction and quantitative PCR were then carried out using miRNA analysis kits specific for each individual miRNA (TaqMan® Real-Time PCR Master Mixes) according to the manufacturer's protocol. The expression level of the U6 gene (ID: 001973) was used as the endogenous control. The primer probe of rno-miR-199a-3p (ID: 002304), rno-miR-181a (ID: 001181), rno-miR-140-3p (ID: 002223) and rno-miR-27b (ID: 000398) were used. All of the quantifications were performed with Applied Biosystems StepOnePlus for 30 mins at 16°C, 30 mins at 42°C, and 5 mins at 85°C, and held at 4°C.

## SUPPLEMENTARY MATERIALS TABLES










