# Supplementary material for: Next-generation sequencing identifies articular cartilage and subchondral bone miRNAs after ESWT on early osteoarthritis knee
Source: Oncotarget. 2016 Aug 17;7(51):84398–407. doi: 10.18632/oncotarget.11331 (PMC5356668; doi:10.18632/oncotarget.11331)
Supplement: Supplementary file 1 [file oncotarget-07-84398-s001.pdf]

Next-generation sequencing identifies articular cartilage and subchondral bone miRNAs after ESWT on early osteoarthritis knee

SUPPLEMENTARY TABLES

Supplementary Data S1: The profiles and expressions of miRNA (up regulation) and gene (down regulation) in articular cartilage with comparisons between sham and OA, sham and OA+ESWT, OA and OA+ESWT.

See Supplemnentary File 1

Supplementary Data S2: The profiles and expressions of miRNA (down regulation) and gene (up regulation) in articular cartilage with comparisons between sham vs OA, sham vs OA+SW, and OA vs OA+SW.

See Supplemnentary File 2

Supplementary Data S3: The profiles and expressions of miRNA (up regulation) and gene (down regulation) in subchondral bone with comparisons between sham vs OA, sham vs OA+SW and OA vs OA+SW.

See Supplemnentary File 3

Supplementary Data S4: The profiles and expressions of miRNA (down regulation) and gene (up regulation) in subchondral bone with comparisons between sham vs OA, sham vs OA+SW, and OA vs OA+SW.

See Supplemnentary File 4
